# ExonMiner: Web service for analysis of GeneChip Exon array data

**DOI:** 10.1186/1471-2105-9-494

**Published:** 2008-11-26

**Authors:** Kazuyuki Numata, Ryo Yoshida, Masao Nagasaki, Ayumu Saito, Seiya Imoto, Satoru Miyano

**Affiliations:** 1 Human Genome Center, Institute of Medical Science, University of Tokyo, 4-6-1 Shirokanedai, Minato-ku, Tokyo, 108-8639, Japan; 2 Institute of Statistical Mathematics, Research Organization of Information and Systems, 4-6-7 Minami-Azabu, Minato-ku, Tokyo, 106-8569, Japan

## Abstract

**Background:**

Some splicing isoform-specific transcriptional regulations are related to disease. Therefore, detection of disease specific splice variations is the first step for finding disease specific transcriptional regulations. Affymetrix Human Exon 1.0 ST Array can measure exon-level expression profiles that are suitable to find differentially expressed exons in genome-wide scale. However, exon array produces massive datasets that are more than we can handle and analyze on personal computer.

**Results:**

We have developed ExonMiner that is the first all-in-one web service for analysis of exon array data to detect transcripts that have significantly different splicing patterns in two cells, e.g. normal and cancer cells. ExonMiner can perform the following analyses: (1) data normalization, (2) statistical analysis based on two-way ANOVA, (3) finding transcripts with significantly different splice patterns, (4) efficient visualization based on heatmaps and barplots, and (5) meta-analysis to detect exon level biomarkers. We implemented ExonMiner on a supercomputer system in order to perform genome-wide analysis for more than 300,000 transcripts in exon array data, which has the potential to reveal the aberrant splice variations in cancer cells as exon level biomarkers.

**Conclusion:**

ExonMiner is well suited for analysis of exon array data and does not require any installation of software except for internet browsers. What all users need to do is to access the ExonMiner URL . Users can analyze full dataset of exon array data within hours by high-level statistical analysis with sound theoretical basis that finds aberrant splice variants as biomarkers.

## Background

It is reported that some splicing isoform-specific transcriptional regulations are related to disease [1,2]. To find disease specific transcriptional regulations, detection of disease specific splice variations is the first step. However, conventional microarrays that produce gene-level information are not suitable for this purpose. On the other hand, Affymetrix Human Exon 1.0 ST Array can measure exon-level expression profiles that are suitable to find differentially expressed exons in genome-wide scale. Affymetrix exon array can measure the transcript levels of more than 1,000,000 exons with 300,000 transcripts by about 6,500,000 probes.

We have developed a supercomputer-based web service named ExonMiner to analyze exon array datasets for detecting genes that are spliced into different isoforms in two types of cells in comparison, e.g. normal and cancer cells. There are some noncommercial standalone applications for analyzing exon array data: IGB [3] is an application for visualizing exon array data and ExACT [4] and Affymetrix Expression Console [5] are mainly focusing on normalizing exon array data. Also, Bioconductor [6] (exonmap [7]) focuses on annotation as well as normalization. The advantage of exonmap is that users can use other statistical tools implemented on R. These are well organized applications, however, these applications focus on data normalizations and we need to use other software for further analysis. Since ExonMiner is, however, an all-in-one web service on a supercomputer system, users can analyze more than 300,000 transcripts spotted on exon array by data normalization, two-way ANOVA analysis, visualization of the results, and detection of exon-level biomarkers. Based on our experiments, which used colon cancer exon array data that contains 20 exon arrays, on various situations of our system usages, the minimal computational time is four hours and the longest was finished in one day. We also observed that the average computational time of colon cancer example is about eight hours.

We have implemented ExonMiner on our Super Computer System  in Human Genome Center, Institute of Medical Science, University of Tokyo and created GUI to use the all analysis tools of ExonMiner easily. An illustrative example of colon cancer exon array data analysis [8] is shown in the web site. ExonMiner has five advantages: (1) a statistical analysis framework, (2) analysis for all transcripts completed, (3) effective visualization with heatmap and barplot images, (4) sophisticated and easy-to-use web interface, and (5) useful hyperlinks to major public databases, e.g. PubMed and NetAffx.

As shown in latter sections, the method implemented in ExonMiner requires more computational time than other software, due to the nonparametric test based on bootstrapping. For example, we need to repeat bootstrap sampling more than 1,000 times for computing accurate *p*-values of statistical tests finding aberrant splice variations, it requires 1,000 times computation of usual statistical test of ANOVA with Gaussian error model. Therefore, we need high-performance parallel computing on Super Computer System. Also, more advanced methods implemented on ExonMiner in future possibly requires more computational resources, therefore, the use of Super Computer System can give flexible computational basis and is suitable for our purpose.

### Data normalization

Before performing statistical analysis, we apply normalization method to raw exon array data. ExonMiner can remove a bias related to GC-content in each probe. The probes are categorized according to their GC-contents and GC-content specific bias will be removed from the probes in each category. ExonMiner uses two types of control for data normalization: One is the median value for each GC category and the other is based on antigenomic background probes. The antigenomic background probes are also categorized into GC categories and we compute their median values. The median value of the probe intensities in each GC category will be transformed by subtracting corresponding control value. In case that user chooses the median values of GC categories for control, the median of probe intensities in a GC category will be equal to one.

### Two-way Analysis of Variance

#### Concept and Model

For using ExonMiner to detect aberrant splice variations, user needs to prepare at least two exon array data from a pair of cells. For example, in our illustrative example, one exon array is prepared for measuring exon profiles in colon cancer cell and the other exon array is used for normal cell. In this case, we can find aberrant splice variants in colon cancer by comparing with normal cells. In this purpose, we use two-way analysis of variance (ANOVA). Suppose that a gene (transcript cluster) is composed of the *m *exonic regions (exon clusters), and that *x*_*ijk *_is the background corrected probe intensity for the *k *th probe (*k *= 1, ⋯, *n*_*ij*_) on the *i *th exon (*i *= 1, ⋯, *m*) of a transcript, i.e. this transcript has *m *exonic regions and each exonic region is spanned by *n*_*ij *_probes. Here the index *j *denotes the type of cells, e.g. *j *= 1 denotes normal cell and *j *= 2 for cancer cell. If we observe *x*_*ijk *_≈ *c *for any *i*, *j *and *k*, the transcript does not show any transcriptional changes and splicing variations across cell types (*j *= 1, 2). If we observe that *x*_*i*1*k *_≈ *c*_1 _and *x*_*i*2*k *_≈ *c*_2 _(*c*_1 _≠ *c*_2_) for any *i *and *k*, it indicates that this transcript was differentially expressed between two cells and this information is equivalent to usual microarray expression data like cDNA microarray, GeneChip and so on. On the other hand, *x*_*ijk *_≈ *c*_1 _and *x*_*i*'*jk *_≈ *c*_2 _for any *j*, *k *and *i *≠ *i' *hold where *c*_1 _≠ *c*_2 _and *c*_1_, *c*_2 _> 0, it means that this transcript has splice variations but these splice variations are commonly occurred between cell types. Finally, if we observe that two cells show different splice patterns, we define them aberrant splice variations. We will capture this information by two-way ANOVA model. For ANOVA in exon array data analysis, see also [8-10].

For detecting transcripts that show aberrant splice variations, we use two-way ANOVA model defined by

**Figure 1:**
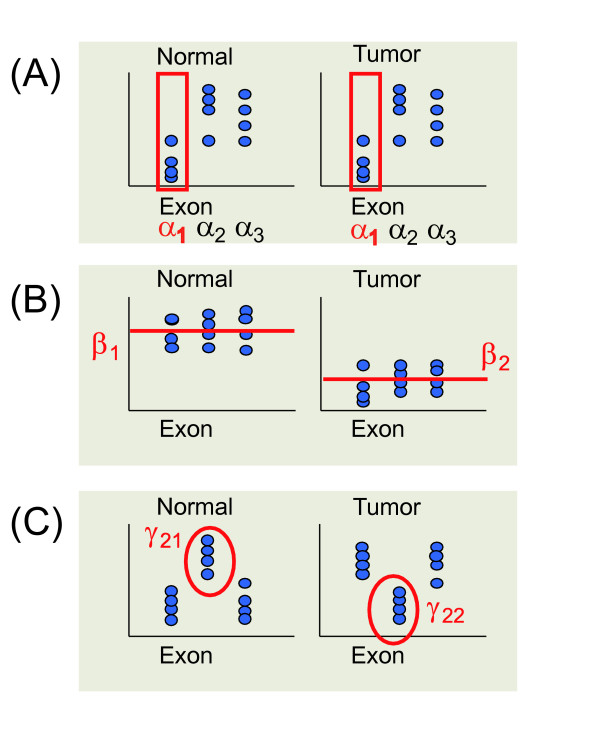
The effects of two-way ANOVA parameters.

*x*_*ijk *_= *μ *+ *α*_*i *_+ *β*_*j *_+ *γ*_*ij *_+ *δ*_*ijk*_,

where *α*_*i*_, *β*_*j *_and *γ*_*ij *_are parameters, *ε*_*ijk *_denotes the observational noise having zero mean and variance *σ*^2^, and *μ *represents an overall mean of the probe intensities. The parameter *α*_*i *_represents the baseline intensities in the *i *th exonic region (*i *= 1, ⋯, *m*), this parameter captures exon effect. The parameters *β*_*j *_(*j *= 1, 2) capture difference in the overall means between two cells, this difference is called overall gene effect. The *γ*_*ij *_s represent interaction effects for each combination of *m *exons and cell types, which is called effect of specific splice variations. The effects of these parameters are shown in Figure 1. A given statistical evidence that one or more *γ*_*ij *_s are different with the others suggests that alternative splicing is present in a particular cell, but absent in the other. We should note that MIDAS [11] is a similar method that uses ANOVA model to analyze exon array data, but MIDAS uses exon-level summarized data, while our model uses probe-level data. Also nonparametric test based on bootstrap method can be considered our advantage.

#### Statistical tests for detecting alternative splicing, differentially expression, and aberrant splice variations

The estimates of *γ*_*ij *_s could capture presence of aberrant splice variations. By the ANOVA model, the probe fluctuations are decomposed into three orthogonal effects, i.e. exon effect (*α*_*i*_), overall gene effect (*β*_*j*_) and effect of specific splice variations (*γ*_*ij*_). The statistical significance of each effect can be evaluated by the following three tests:

**Test 1 **(Detection for exon effect):

H_0_: *α*_*i *_= 0 for all *i*.

H_*a*_: *α*_*i *_≠ 0 at least one *i*.

**Test 2 **(Detection for overall gene effect):

H_0_: *β*_1 _= *β*_2_

H_a_: *β*_1 _≠ *β*_2_

**Test 3 **(Detection for effect of specific splice variations):

H_0_: *γ*_*ij *_= 0 for all *i *and *j*.

H_a_: *γ*_*ij *_≠ 0 for one or more pairs of (*i*, *j*).

Here H_0 _and H_a _represent null and alternative hypotheses, respectively. Repeating these hypothesis tests for all transcript clusters, one can obtain the statistical evidences of aberrant splice variations which are scored by the computed *p*-values from Test 3. In ExonMiner, in addition to the usual F-test for test of parameter significance, the permutation method that is a nonparametric approach is developed to calculate the null distribution of the F-statistics; *F*_exon_, *F*_gene _and *F*_sas_, for assessing significance of exon effect, gene effect and effect of specific splice variations, respectively. In order to evaluate the null distributions, we first generate a permutation set of samples by bootstrapping *n *= Σ_*ij*_*n*_*ij *_samples from *x*_*ijk *_s. Repeating this process *B *times, we can approximately evaluate the null distribution of each F_* _with the *Q *permutation statistics $f_{0^{*}}^{\left(q\right)}$, *q *= 1, ⋯, *Q *. Note that * can be replaced by exon, gene and sas. Subsequently, the *p*-value for a given test statistic F_* _= f_* _obtained from the original data set is calculated by

$$p_{*}=\frac{\#\{q:f_{0^{*}}^{\left(q\right)}\geq f_{*}\}}{Q}$$

for each effect. In ExonMiner, users can choose parametric or nonparametric test for assessing significance of each parameter.

### Meta Analysis

To detect aberrant splice variants as biomarkers, we need to check whether the detected aberrant splice variants are common in the targeted disease or not. In this purpose, we establish a statistical testing procedure based on meta-analysis [8]. Suppose that we have *G *pair of exon array datasets, i.e. normal and tumor exon expression data are measured from *G *patients. By performing the whole transcript analysis based on two-way ANOVA to *G *paired exon array datasets, one obtains a set of *p*-values for each effect, e.g. effect of specific splice variations (*γ*_*ij*_), $p_{1}^{g},\cdots ,p_{r}^{g}$, across patients, *g *= 1, ⋯, *G*. Here the total number of transcripts analyzed is denoted by *r*. Intuitively, a transcript having a small *p*-value is strongly associated with tumor formation. However, it is possible that some observed aberrant splice variants could be caused by the inter-individual differences of the analyzed samples. Our goal is to discover the "universal biomarkers", i.e. aberrant splice variations which are shared by most individuals with a particular diagnostic category.

Following this direction, we develop the statistical technique within the framework of meta-analysis based on the normal inversion method.

Let $x_{ijk}^{g}$ denote the observed probe intensities of the *k *th probe which spans the *i *th exonic region for normal cell (*j *= 1) or target cell (*j *= 2) isolated from the *g *th individual. We assume that the probe intensities $x_{ijk}^{g}$ of each individual can be modeled by the two-way ANOVA defined by

$$x_{ijk}^{g}=\mu^{g}+\alpha_{i}^{g}+\beta_{j}^{g}+\gamma_{ij}^{g}+\varepsilon_{ijk}^{g},$$

for *g *= 1, ⋯, *G*. Given these models, the statistical hypothesis testing of each effect, for example, effect of specific splice variations, is formulated by

**Test 4 **(Detection for universal specific splice variations):

H_0_: $\gamma_{ij}^{g}$ = 0 for all *i*, *j *and *k*.

H_a_: $\gamma_{ij}^{g}$ ≠ 0 for one or more tuple (*i*, *j*, *g*).

In order to assess the H_0_, we propose use of the normal inversion metric as a test statistic. Suppose that we have a set of *p*-values, $p_{h}^{1},\cdots ,p_{h}^{G}$, for occurrence of the aberrant splice variations in the *h *th transcript cluster. The method first converts these *p*-values into the *z*-scores as $z_{h}^{g}=\Phi^{-1}(1-p_{h}^{g})$, where Φ^-1 ^is the inversion of the standard normal cumulative distribution function, and then computed integrated *z*-score as

$$z_{h}=\frac{\sum_{g=1}^{G}z_{h}^{g}}{\sqrt{G}}.$$

The significance of H_a _can be assessed based on the integrated *p*-value which is computed by transforming the *z*-score with the standard normal cumulative distribution function Φ as

$$p_{h}^{\text{integrated}}=1-\Phi (z_{h}).$$

We would like to show an actual example of meta-anlaysis. In Yoshida *et al*. [8], colon cancer exon array dataset was analyzed by primary version of ExonMiner. In this anlaysis, based on the Test 3 of ANOVA, gene *MUC17 *(Accession ID: NM_001040105) has *p*-values for ten patients:

*p*_*h*_^1 ^= 0.313; *p*_*h*_^2 ^= 0.0005; *p*_*h*_^3 ^= 0.0005; *p*_*h*_^4 ^= 0.8964; *p*_*h*_^5 ^= 0.8201;

*p*_*h*_^6 ^= 0.0002; *p*_*h*_^7 ^= 0.6549; *p*_*h*_^8 ^= 0.0179; *p*_*h*_^9 ^= 0.0522; *p*_*h*_^10 ^= 0.1664.

These p-values are transformed into z-scores as:

*z*_*h*_^1 ^= 0.487; *z*_*h*_^2 ^= 3.291; *z*_*h*_^3 ^= 3.291; *z*_*h*_^4 ^= -1.261; *z*_*h*_^5 ^= -0.916;

*z*_*h*_^6 ^= 3.540; *z*_*h*_^7 ^= -0.399; *z*_*h*_^8 ^= 2.099; *z*_*h*_^9 ^= 1.624; *z*_*h*_^10 ^= 0.968.

The integrated *z*-score is 4.023 and the integrated *p*-value is obtained as 2.86 × 10^-5^.

In the colon cancer example, we compute *q*-values from integrated *p*-values of meta-analysis, the list of the genes identified as having aberrant splice variations including exon skipping/retaining has 10% False Discovery Rate (FDR) that corresponds to *q*-value < 0.1. In the above *MUC17*, the *q*-value is 0.0345 and it is determined as significant. The computation of *q*-value is shown in Yoshida *et al*. [8].

By using exon array data with ExonMiner, it is possible to detect alternative splicing like exon skipping/retaining, alternative usage of donor and acceptor splice sites and so on. However, since exon array does not have junction probes, custom array with junction probes or PCR method are needed for further analysis of detecting exact patterns of splice isoforms.

## Implementation

### Data upload

The users are required to upload their exon array data. We prepared an FTP service for data upload. A reason for choosing FTP service for our system is that a large dataset can easily be uploaded. To increase the security level, we prepare one time account and password for FTP service. Note that one time account and password are different from the pair for login account and password of ExonMiner.

### Statistical analysis engine

ExonMiner performs ANOVA for each transcript. To test the significance of each effect in ANOVA described in previous section, we implemented two types of tests: one is based on Gaussian noise model and it performs F-test, the other is based on nonparametric approach using bootstrap method. In the nonparametric approach, we need to compute test statistics repeatedly and it needs enormous computation. Therefore we implemented the ANOVA program by Fortran and optimized for high performance computing described in the latter section.

### Visualization engine

The information of exon expression pattern for each transcript needs to be shown visually. We have developed two types of image generators and can make heatmap and barplot images optimized for exon array data. These images are generated by using R. The graphics library is originally developed.

### Database

For the management of user information and probe annotation information, we use MySQL database server. For constructing a highly secure system, user login information is encrypted and stored in MySQL database. By keeping probe annotation information into MySQL database, users are not necessary to explore other databases. Thus ExonMiner is an all-in-one web service.

### High performance computing on supercomputers

Since ANOVA for the full set of transcripts needs high performance computing, we perform each ANOVA computation in parallel on our supercomputer system. Our supercomputer system has eight Sun Fire 15 k and at most 700 CPUs can be used for parallel statistical computation by using Sun Grid Engine.

### Web interface

In ExonMiner, PHP scripts deal with connections between front end users and our supercomputer system and dynamically generate images by executing visualization engine described above based on user input. PHP scripts generate HTML web pages with a uniformed style that increases usability.

## Results and discussions

### Overview of ExonMiner

#### Create user account

**Figure 2:**
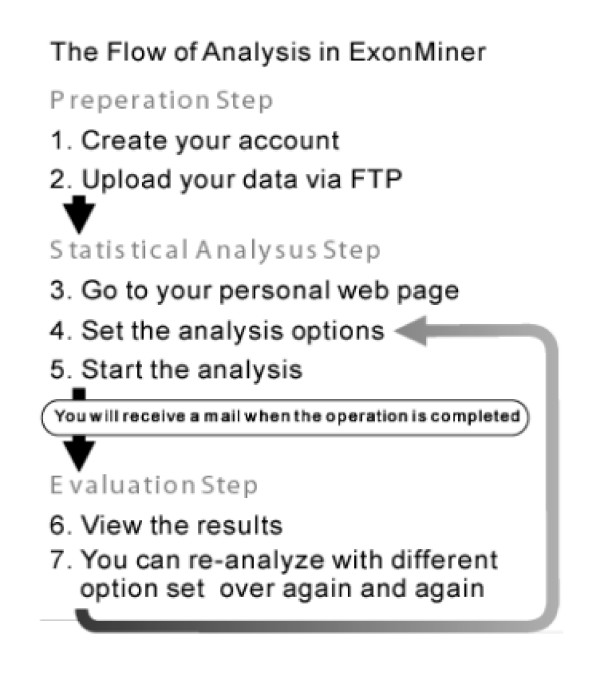
A flowchart for an analysis in ExonMiner.

**Figure 3:**
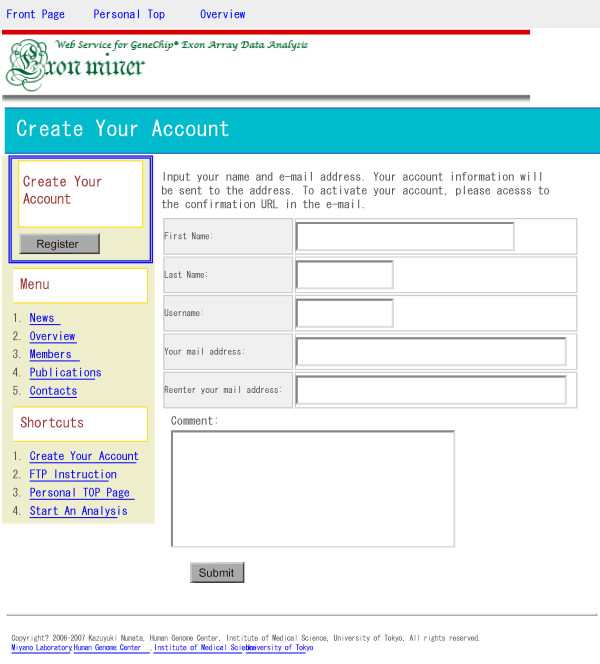
A snapshot of the account registration screen.

Figure 2 shows a flowchart of ExonMiner. First, a user account will be created by request to ExonMiner. Figure 3 shows the web page for user account registration. By filling the registration form, an e-mail with (1) ID (username), (2) login password and (3) confirmation URL will be sent to the user. Accessing the confirmation URL, the user ID will be activated and the personal page for the user is dynamically created.

#### FTP for data upload

For the upload of your data, you need to use FTP. For using FTP service in ExonMiner, user needs to get one time password and account for FTP.

Note that the account of FTP is different from login account. Using the one time password, the user can upload CEL (TEXT) files archived by ZIP via FTP. ExonMiner supports CEL files as TEXT format (this CEL file is recognized as version 4), usual CEL files are, however, BINARY format (this CEL file is recognized as version 3). To convert BINARY CEL files (version 3) in TEXT format (version 4), "*CEL File Conversion Tool*" provided by Affymetrix Inc. is available [12].

#### Analysis options

**Figure 4:**
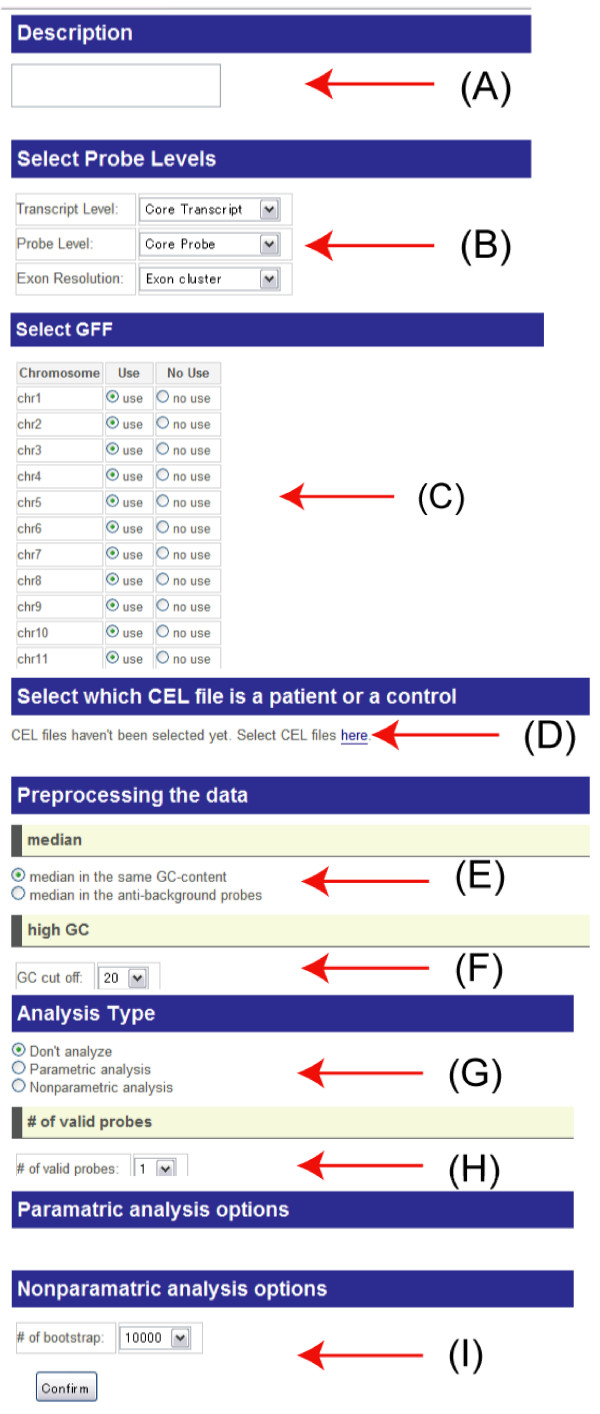
A snapshot of the analysis option selection screen.

User should fill up all of the analysis options. Then user will start the analysis. User must select all (A) – (I) options in Figure 4 to start a statistical analysis by two-way ANOVA and meta-analysis.

(A) Description: you can add a brief description of your analysis. It may be convenient that you put a name of this analysis to organize your analyses.

(B) Select probe levels: you can select the level of expression information in exon array. Transcript Level: For transcripts, there are three levels, core, extended and full transcripts, according to their information quality based on their information sources. Like transcript level, user can choose Probe Level and Exon Resolution.

(C) Select GFF: you can select chromosomes. Transcripts on the selected chromosomes will be analyzed. This selection can reduce computational time.

(D) Select which CEL file is a patient or a control: user adds the outcome information to each CEL file you have uploaded by FTP.

(E) Preprocessing data (background correction): user selects the type of normalization method. GC-content: the median values in the same GC-content probe groups are used as control values. Antigenomic background: the median values in the same GC-content antigenomic background probes are used as control values.

(F) Preprocessing data (GC-content threshold): it is a possible case that probes with high GC-content work as noise. So you can remove such probes. In default, the probes with 20 or more GC-content are removed. If you want to use the all probes for analysis, you choose 26 as the cut-off.

(G) Analysis type (model): user selects the analysis type from the following three types – Don't analyze: ExonMiner does not perform ANOVA. Only visualization and sequence information are available. Parametric analysis: Gaussian distribution is assumed as the noise model. Nonparametric analysis: ExonMiner does not assume any distributions for the noise model. Bootstrap test will be applied for computing *p*-values.

(H) Analysis type (threshold for the number of probes): ExonMiner ignores probesets or exon clusters with small number of probes for stabilizing the results of ANOVA. You can choose this cut-off by this option.

(I) Nonparametric analysis options: the number of bootstraps in nonparametric ANOVA is specified by this option.

#### Visualization of the results

**Figure 5:**
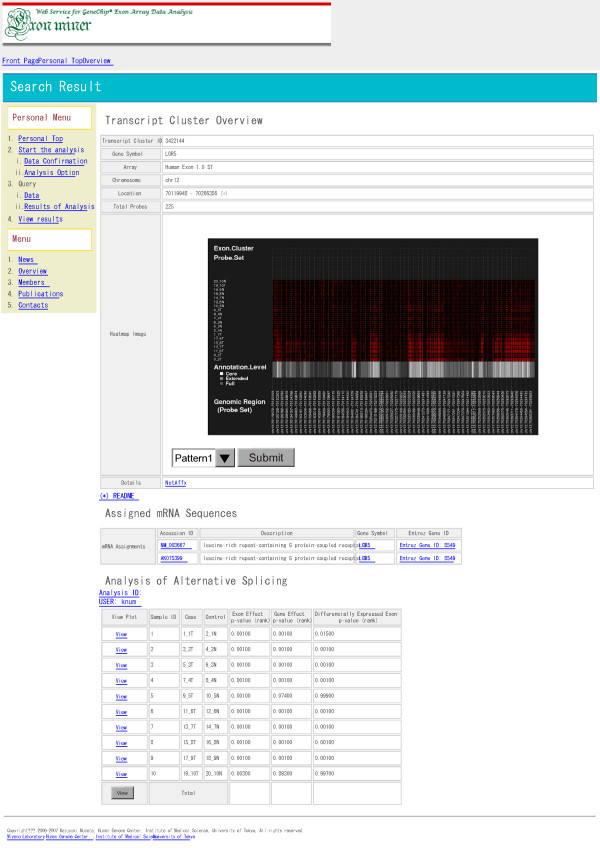
A snapshot of the analysis result viewer.

Setting the all options, user can start the analysis. When the analysis is completed, ExonMiner sends an e-mail to the user to announce that the calculation is finished. After that, the user can view result pages of the analysis with heatmaps, barplots, sequence information and calculated *p*-values of two-way ANOVA and results of meta-analysis. A screen shot of ExonMiner is given in Figure 5. In this figure, you can see the results of LGR5. LGR5 is one of the most significant genes in colon cancer exon arrays reported by Yoshida *et al*. [8]. The colon cancer exon array data are provided by Affymetrix. We can reach the information for each transcript by either gene symbol or transcription cluster ID. The heatmap (A) represents the exon profiles of LGR5. The user can download the heatmap image as bitmap or postscript file. Sequence information (B) for the transcript is shown with hyperlinks to the external web sites, Entrez [13] and NetAffx [14]. The table (C) shows calculated ANOVA *p*-values. User can view the barplot image of normalized exon expression for a pair of cells from the View hyperlinks. The *p*-values for parameters calculated in meta-analysis are shown in the bottom table. The user can download results in one Excel file.

**Figure 6:**
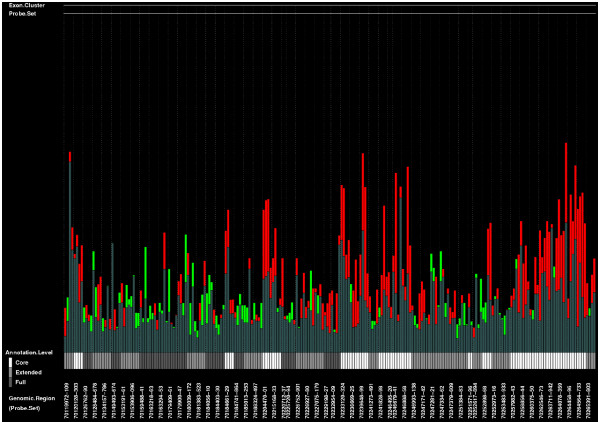
A sample barplot image for each exon expression level.

Instead of the heatmap image, ExonMiner can produce barplot images. Figure 6 is a barplot image for LGR5. A barplot image has three bar-graphs. Red bar-graph shows probe intensities in exon array of colon cancer cell and green bar-graph shows probe intensities in exon array of normal cell. We show the bars with lower intensities in dark color. If the color of the bar on a dark bar is red, the cell type of the dark bar is normal (green) and *vice versa*. By using dark bar-graph, the users easily find the differences of exon expressions between two cells. For example, from Figure 6, we can find that the exon expression levels of colon cancer cell are higher than those of normal cell in many exonic regions.

## Availability and requirements

• Project name: ExonMiner

• Project home page: 

• Anonymous accounts (no e-mail address for registration is needed): 

• Operating systems: any OS (that has an internet browser application)

• Programming language: PHP, R, Fortran, Perl, Ruby, MySQL

## Conclusion

ExonMiner is an all-in-one web service well suited for analysis of exon array data. Since it does not require any installation of software except for internet browsers, what all users need to do is to access the ExonMiner URL . ExonMiner can perform not only visualization of exon array data, but also can perform data normalization and user customized statistical analysis that is hard to run on a single computer. With the support of supercomputers in Human Genome Center, Institute of Medical Science, University of Tokyo, users can analyze full dataset of exon array data within hours with results of meta-analysis that finds aberrant splice variants as biomarkers.

## Authors' contributions

KN, AS and MN designed ExonMiner and KN implemented. KN and AS prepared the figures. RY and SI developed statistical analysis in ExonMiner. SM supervised the project. KN wrote the manuscript.
